# Bioelectrical impedance vector analysis and phase angle in boys with Duchenne muscular dystrophy

**DOI:** 10.29219/fnr.v63.1615

**Published:** 2019-04-10

**Authors:** Karina M. Vermeulen, Márcia M. G. D. Lopes, Evellyn C. Grilo, Camila X. Alves, Richele J. A. Machado, Lucia L. Lais, José Brandão-Neto, Sancha H. L. Vale

**Affiliations:** 1 Postgraduate Program in Health Sciences, Federal University of Rio Grande do Norte, Natal, Brazil; 2 Department of Nutrition, Federal University of Rio Grande do Norte, Natal, Brazil; 3 Departament of Internal Medicine, Federal University of Rio Grande do Norte, Natal, Brazil

**Keywords:** Duchenne muscular dystrophy, phase angle, bioelectrical impedance vector analysis, body composition, lean mass

## Abstract

**Background:**

Duchenne muscular dystrophy (DMD) is a disease characterized by progressive loss of functional muscle mass followed by changes in body composition.

**Objective:**

This study aimed to describe and evaluate bioimpedance parameters in boys with DMD.

**Design:**

This cross-sectional, descriptive study investigated children and adolescents diagnosed with DMD. Age, weight, height, resistance, and reactance data were collected. Phase angle and bioelectrical impedance vector analysis were calculated based on resistance and reactance values.

**Results:**

We analyzed 43 boys aged between 2.7 and 19.8 years. Low-phase angle values were observed during the investigation of bioimpedance parameters. Bioelectrical impedance vector analysis showed that approximately 87% of the subjects presented vectors outside the tolerance ellipses, and only one patient presented vectors located within the 50% tolerance ellipse, indicating normally hydrated and a good body cell mass. Compared with the reference population, boys with DMD had lower levels of body cell mass.

**Conclusion:**

Based on the evidence, compared with the reference population, patients with DMD had lower levels of body cell mass. This evidence points to bioimpedance parameters as useful tools for the nutritional evaluation and clinical management of patients with DMD.

## Popular scientific summary

- Duchenne muscular dystrophy (DMD) is a neuromuscular disease that causes irreversible degeneration of the muscle tissue, leading to nutritional complications that worsen with age.
- Bioelectrical parameters were evaluated for the first time in patients with DMD and differed from those reported in healthy and ill subjects of the same age.
- This evidence points to bioimpedance parameters as useful tools for the nutritional evaluation and clinical management of patients with DMD.

Duchenne muscular dystrophy (DMD) is a severe degenerative neuromuscular disease displaying X-linked recessive inheritance and affects approximately 1 in 3,500 to 5,000 live male births; Hispanic individuals exhibit the highest specific prevalence (1, 2). DMD is caused by mutations in the gene that encodes the dystrophin protein (Xp21.2), which leads to a deficiency in this protein, resulting in irreversible degeneration of the muscle tissue. This condition is characterized by a progressive loss of lean mass and muscle strength, leading to severe disability and, if untreated, early death in late adolescence (3, 4).

Nutritional complications are present in patients with DMD and worsen with age (5). Specifically, as the child grows, nutritional aspects of the disease must be considered, such as changes in body composition, with an increase in fat mass and a decrease in lean mass, as well as the deleterious effects of malnutrition on glucose metabolism, respiratory and cardiac functions, and mobility (5–7).

The nutritional status of a patient with DMD is strongly influenced by the progression of the disease and the side effects of drug therapy (8). In addition, the application of some nutritional assessment methods, such as body mass index (BMI) and skinfold measures, is imprecise. The reason is that the measurement techniques and reference range applicable to the general population are inadequate for the population with DMD, making it difficult to interpret the markers used in the nutritional evaluation of these patients (6).

Bioelectrical impedance analysis (BIA) is a good method that can be used to evaluate the body composition of a diverse population. This method is based on the principle that body components behave as an electric circuit in a steady state, offering an opposing force to an electric current when it is applied to the circuit. Impedance, another name for this opposing force, comprises two vectors: resistance (R) and reactance (Xc). The phase angle (PA) is calculated from these vectors, enabling researchers to perform bioelectrical impedance vector analysis (BIVA) (9, 10).

The PA reflects the different electrical properties of body tissues that are affected by the disease, nutritional status, and hydration. Additionally, it reflects changes in the electrical conductivity of the body, indicating alterations in the integrity of cell membranes and intercellular space (9–12). Thus, this parameter is useful for assessing disease severity as well as patient prognosis in different clinical settings. For these reasons, PA seems to be superior to other nutritional, anthropometric, and serum parameters (13).

BIVA allows a patient to be evaluated through direct measures of vector impedance, which represents a more reliable evaluation of body composition without the need of equations or models that are primarily affected by an individual’s biological variables. BIVA has been used to evaluate hydration state and cell mass, providing a graphical method for representing R and Xc corrected for body length based on analysis of the bivariate distribution of vector impedance in a healthy population with specific characteristics (9).

Nutritional care should begin from the moment of diagnosis and must be available as needed (14). New parameters that evaluate nutritional prognosis must be analyzed because previous studies have not examined bioelectric parameters in patients with DMD. Therefore, the aim of this study was to describe and analyze the bioelectric parameters in individuals with this disease.

## Methods

### Study population

A cross-sectional, nonprobability sampling, descriptive study was performed after receiving approval from the ethics and investigation in humans committee and was registered in the Brazilian Register of Clinical Trials (RBR-7cfdxm). Each participant and their guardians signed the consent form.

Sampling included all patients with DMD under 20 years of age treated at the neurology outpatient facility at the Onofre Lopes University Hospital, Brazil, who were attended by the nutrition team between September 2016 and December 2018 and underwent BIA. The patients were grouped according to the age range, with Group 1 (G1) including children between 2 and 9 years old and Group 2 (G2) including adolescents between 10 and 19 years old. Boys for whom it was not possible to obtain measures of weight and/or height (*n* = 4) were excluded from the BIVA analysis.

**Fig. 1:**
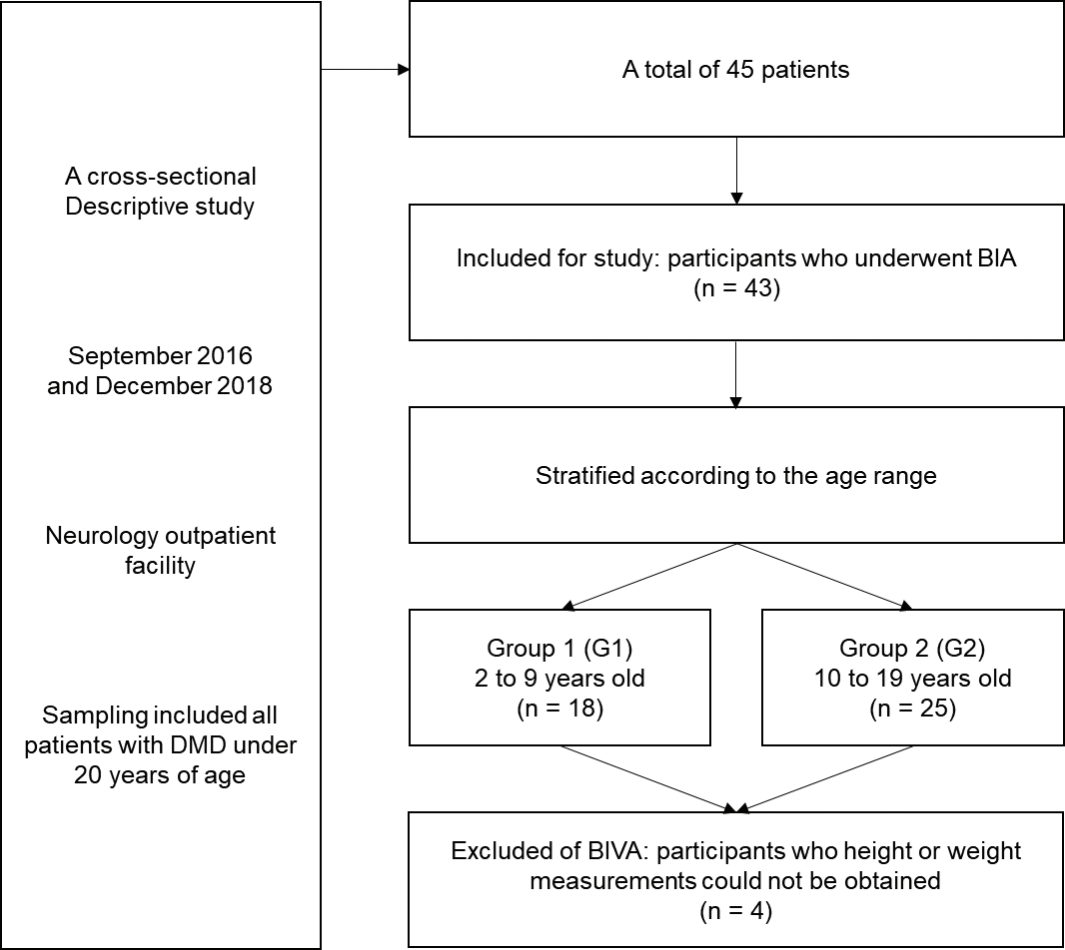
Flowchart of sample recruitment and selection procedures.

The recruitment and selection procedures are described in Fig. 1.

### Anthropometry

Weight (kg) and height (m) of the subjects were measured. A platform-balance (KN Waagen, São Paulo, Brazil) was used to obtain weight measurements, with a ramp to assist patients using wheelchairs. Height was measured with a stadiometer (Sanny Stadiometer Professional, American Medical of Brazil, São Paulo, Brazil) or was estimated using the formula proposed by Stevenson (15), which uses the knee height corresponding to the distance between the knee and ankle, as measured with an inextensible millimeter tape.

BMI was calculated as the ratio between body weight and height squared (showed in kg/m^2^). The weight-for-age (WAZ), height-for-age (HAZ), and BMI-for-age (BAZ) z-scores were calculated using AnthroPlus software v1.0.4 (available at www.who.int/growthref/en/) and were ranked according to the growth curves of the World Health Organization for healthy children aged 2 to 19 years (16). Anthropometric assessments were performed by trained nutritionists.

### Bioelectrical impedance analysis

The bioimpedance parameters R (Ω) and Xc (Ω) were obtained using the Quantum II^®^ body composition analyzer (RJL Systems, Clinton Township, MI, USA) using the passage of a single frequency (50 kHz), which was painless and safe. This whole-body BIA device employed a tetrapolar method that was performed with the subject lying supine. Four self-adhesive spot electrodes were positioned with two electrodes placed on the dorsal surface of the right hand and two electrodes placed on the dorsal surface of the right foot, as described by Lukaski et al. (17). This measurement takes a duration of less than 5 min.

### Bioelectrical impedance vector analysis

R and Xc data were subsequently used to determine the PA and BIVA. The PA was calculated using the following formula: PA = tangent arc (Xc/R)*180/π (18). The BIVA results were based on the analysis of normalized R and Xc values for the patients’ height (H) measurements (R/H and Xc/H in Ω/m), as a single vector measured in an individual at a single time.

Individual measurements were compared to confidence intervals (elliptical shape) determined for the healthy population. The vector shift over the ellipse is a semiquantitative method for assessing body composition, whereas the shortening or lengthening of the vector suggests changes in body hydration. The tolerance ellipses were determined using the BIVA 2002 software developed by Piccoli et al. (19).

**Fig. 2:**
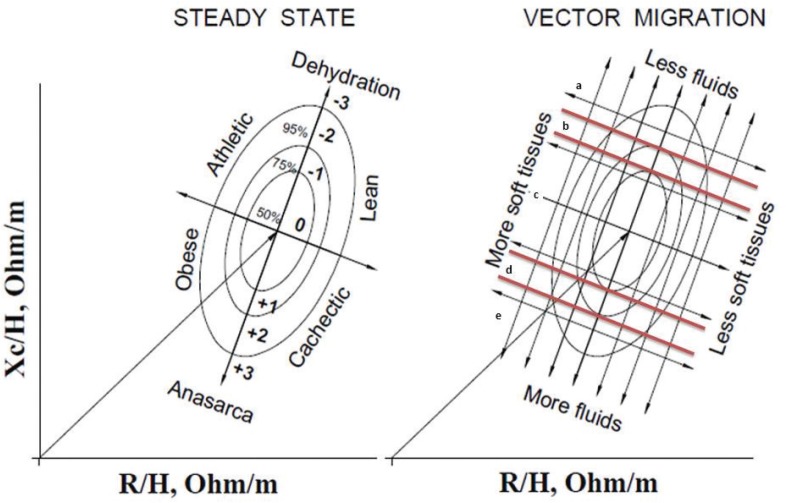
Different trajectories indicate combined changes in both hydration and tissue mass. Classification of hydration and lean mass status using the R/Xc graph and the 50, 75, and 95% percentile tolerance ellipses. These percentiles were used to project a 5-point hydration scale on the BIVA plain. Positions (*a*) and (*b*) represent ‘less hydrated’ individuals; position (*c*) represents ‘normally hydrated’ individuals; and positions (*d*) and (*e*) represent ‘more hydrated’ individuals (20). Adapted from Piccoli and Pastori (21).

Participants’ data were plotted on the R/Xc point graph using the 50, 75, and 95% tolerance ellipses from a healthy population at the same age, as proposed by Piccoli et al. (19). Hydration status was determined by calculating the position of each individual’s baseline bioimpedance vector on the BIVA R/Xc plane. The plane is a five-point graph corresponding with the boundaries of each tolerance ellipse (Fig. 2). Individuals with vectors located in (or above) the 75% tolerance ellipse (positions *a* and *b*) were classified as ‘less hydrated’. Participants with vectors located in the central 50% percentile ellipse (position *c*) were classified as ‘normally hydrated’. Participants with vectors located in (or below) the lower 75% percentile range (positions *d* and *e*) were classified as ‘more hydrated’ (20).

### Statistical analysis

Statistical analysis was performed by observing the distribution of the data in a normal curve using the Shapiro–Wilk test. Quantitative variables with a normal distribution were expressed as the means and standard deviations, and variables that did not meet the Gaussian model were presented as medians and interquartile ranges. Correlations between variables were analyzed using Spearman’s correlation test. A value of *P* < 0.05 indicated statistical significance.

## Results

This study included 43 boys with DMD aged between 2.7 and 19.8 years. Regarding medical status and nutritional support, the children did not present acute diseases, 86% of the total population used corticosteroids, and 88% used calcium and vitamin D supplementation, which are part of the outpatient protocol. Among all the boys assessed, 23.7%were stunted (HAZ <−2), 5.6% were underweight (WAZ< −2),and 19.4% were wasted (BAZ< −2).

**Fig. 3:**
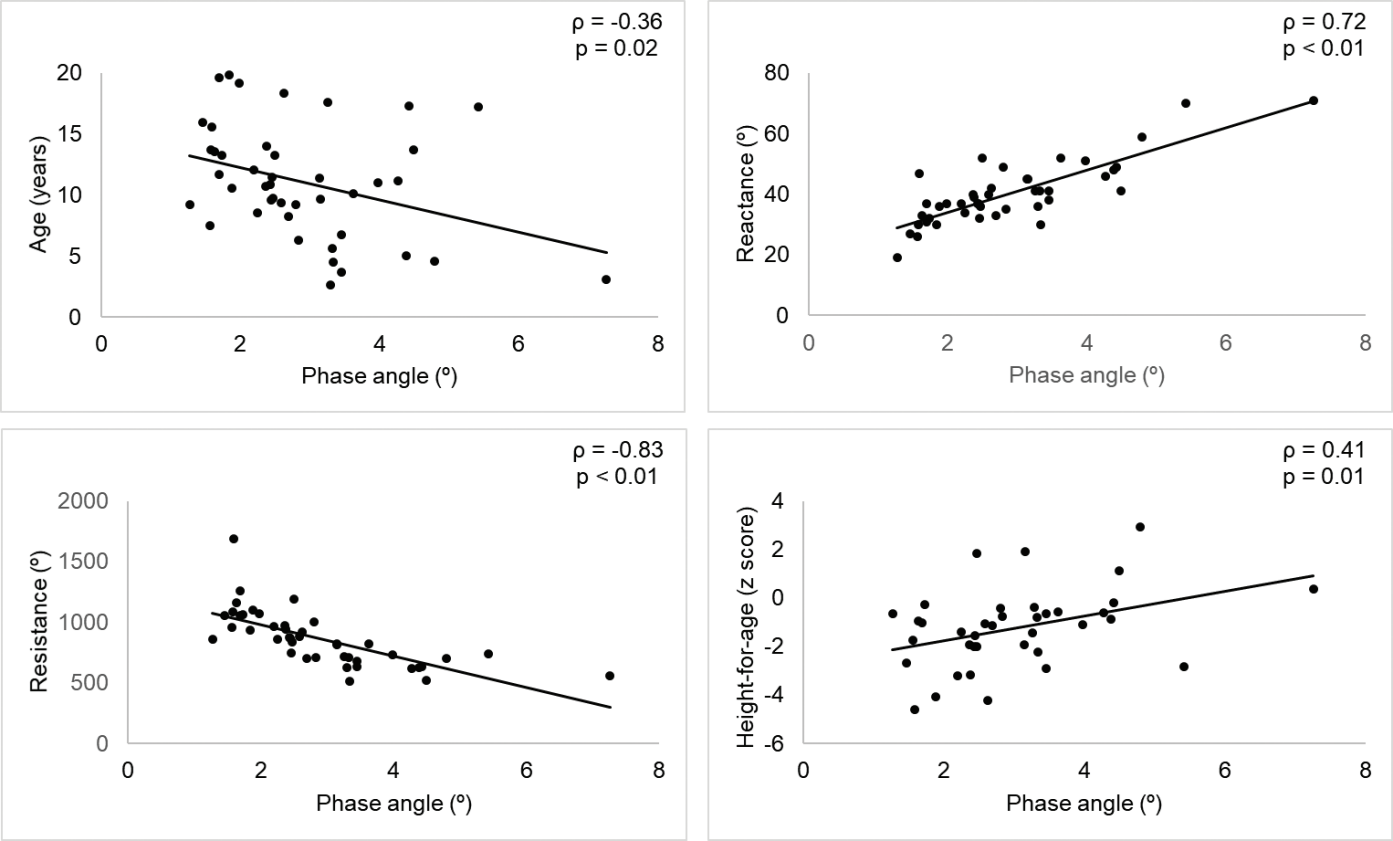
Spearman’s correlation (ρ) analyses between phase angle and age, reactance, resistance, and height-for-age of boys diagnosed with Duchenne muscular dystrophy.

Anthropometric and bioimpedance parameters, as well as PA values, were distributed according to age in Group 1 (2 to 9 years) and Group 2 (10 to 19 years), as shown in Table 1. PA values were correlated only with age, resistance, reactance, and HAZ, as shown in Fig. 3.

**Table 1:** Anthropometric and bioelectrical parameters of boys diagnosed with Duchenne muscular dystrophy distributed according to group

Participants (*n*)	Group 1	Group 2
	18 (42%)	25(58%)
Age (years)	6.9 (2.5)	14.1 (3.2)
	7.1 (5.6; 8.1)	13.6 (12.8; 15.4)
Weight (kg)	24.0 (12.6)	39.9 (18.9)
	20.9 (17.7; 30.2)	34.5 (31.7; 48.1)
Height (*m*)	1.15 (0.15)	1.44 (0.16)
	1.18 (1.07; 1.23)	1.40 (1.37;1.51)
Weight-for-age (*z* score)¹	−0.17 (1.74)	–
	−0.22 (−1.04; 0.70)	–
Height-for-age (*z* score)²	−0.74 (1.40)	−1.69 (1.71)
	−0.83 (−1.43; −0.04)	−1.68 (−2.49; −0.89)
BMI-for-age (*z* score)²	0.58 (1.60)	−0.98 (2.26)
	0.34 (−0.21; 1.38)	−0.64 (−2.10; 0.14)
Resistance (Ω)	753 (138)	948 (246)
	708 (684; 822)	940 (846; 1,050)
Reactance (Ω)	40 (12)	41 (10)
	38 (34;46)	39 (36.6; 44.5)
Phase angle (°)	3.2 (1.3)	2.7 (1.1)
	3.0 (2.5; 3.8)	2.4 (2.2; 3.1)

Group 1: 2 to 9 years old; Group 2: 10 to 19 years old. Continuous variables are presented as the means (standard deviations) and medians (95% CI, lower; upper values).

¹ Classification for individuals aged 2 to 10 years (*n* = 18).

**Fig. 4:**
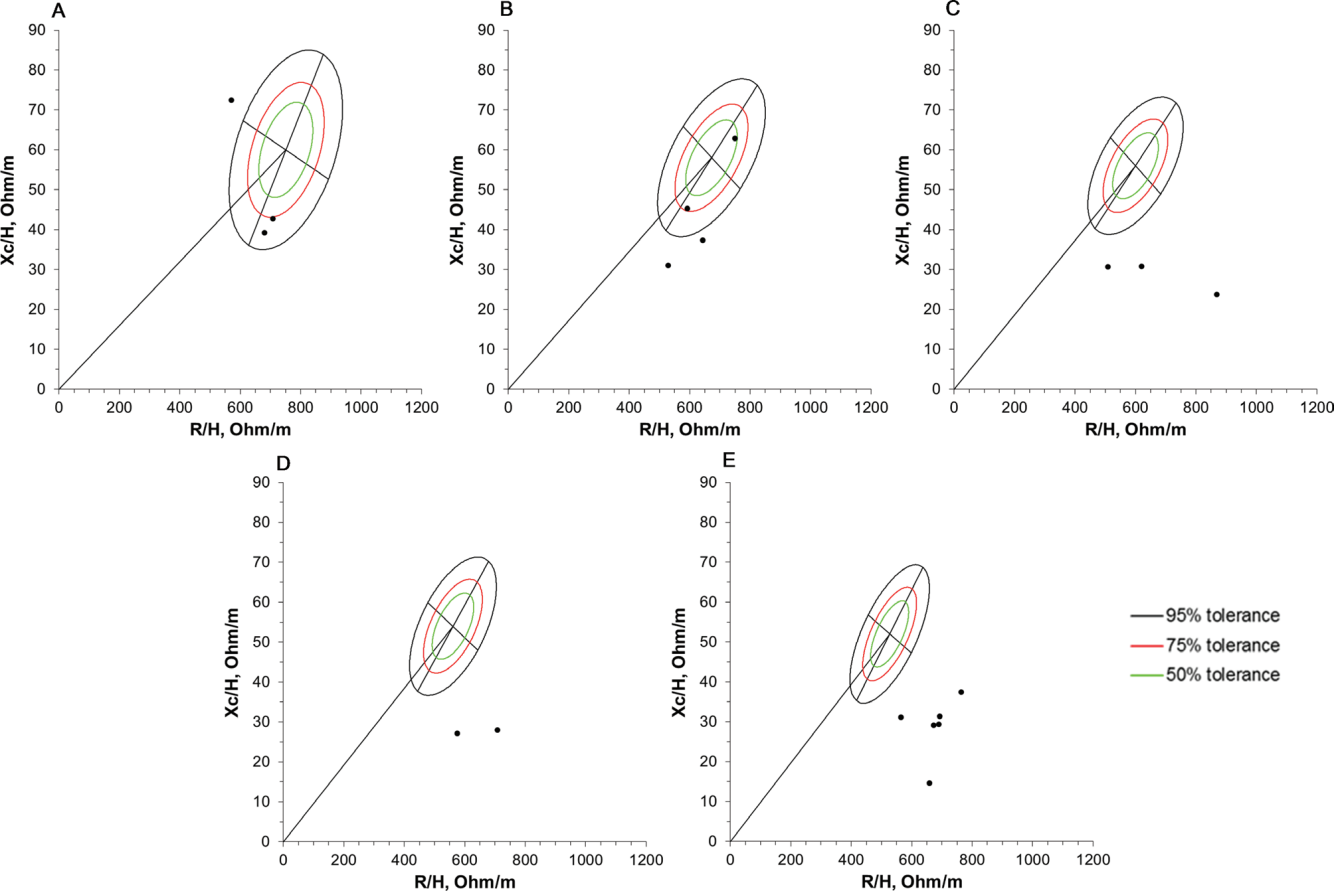
BIVA patterns of 18 boys diagnosed with Duchenne muscular dystrophy. Impedance vectors plotted on the 50, 75, and 95% tolerance ellipses of the corresponding reference population of healthy male children (22). (A) 2 to 3 years old (*n* = 3), (B) 4 to 5 years old (*n* = 4), (C) 6 to 7 years old (*n* = 3), (D) 8 years old (*n* = 2), and (E) 9 years old (*n* = 6). R/H, resistance-height; Xc/H, reactance-height.

**Fig. 5:**
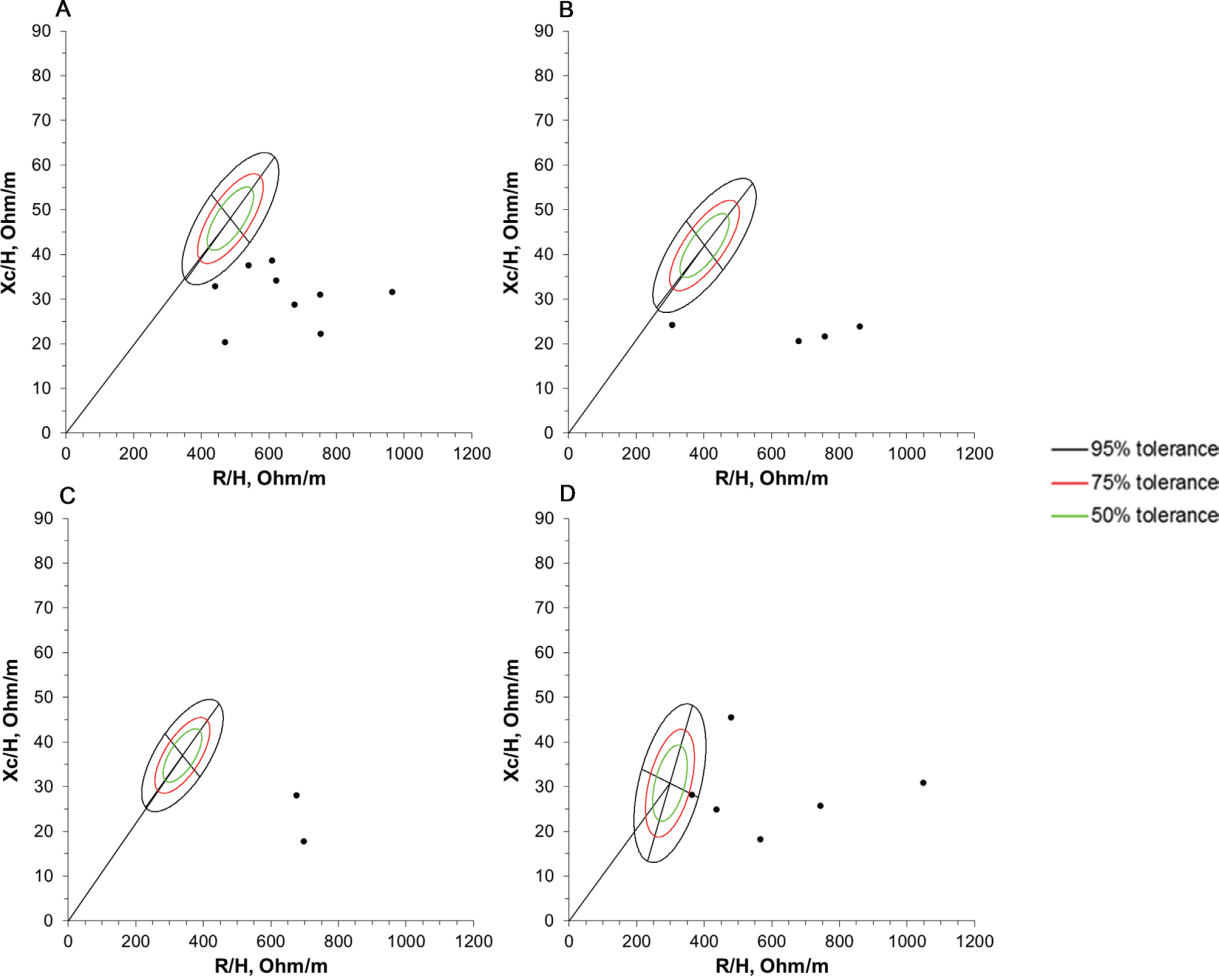
BIVA patterns of 21 boys diagnosed with Duchenne muscular dystrophy. Impedance vectors plotted on the 50, 75, and 95% tolerance ellipses of the corresponding reference population of healthy male children (22). (A) 10 to 11 years old (*n* = 9), (B) 13 years old (*n* = 4), (C) 14 to 15 years old (*n* = 2), (D) ≥ 16 years old (*n* = 6). R/H, resistance-height; Xc/H, reactance-height.

² Classification for individuals aged 2 to 19 years (*n* = 40);

The results from the BIVA are shown in Figs. 4 and 5 for children and adolescents, respectively. Thirty-four of thirty-nine (87.2%) subjects presented vectors outside the tolerance ellipses, and only one participant presented vectors located within the 50% tolerance ellipse.

In the analysis shown in Fig. 4A, one child is located outside of 95% tolerance ellipses of the upper left quadrant, indicating more lean mass than a reference population of the same age. In Fig. 4B, only one participant is located within the 50% tolerance ellipse of the upper right quadrant, indicating normally hydrated and a good body cell mass. In Fig. 4C, D, and E, the boys’ vectors are located in the lower right quadrant from the 95% tolerance ellipses, indicating more hydrated and a lower cellular mass. Fig. 5A, B, C, and D shows heterogeneity in the vector trends comprising less hydrated, more hydrated, and normally hydrated patients, all of whom had low cell masses.

## Discussion

The present study evaluated the anthropometric and bioimpedance parameters of 43 individuals with DMD. The bioimpedance parameters were used to determine the PA and BIVA values, which can show body composition changes even before changes in anthropometric indicators can be detected. Our results showed that PA values in DMD boys were lower than reference values.

The PA is an indicator of change in the integrity of cell membranes and the intercellular space (23). Lopes et al. (24) reported a mean PA of 5.0° in the healthy population. In our study, we observed lower values of 3.0° and 2.4° for G1 and G2, respectively. These values were also lower than the average value reported in a study of seven children with osteogenesis imperfecta, who presented PA values of 4.8° (25).When the data were distributed by age group, lower PA values were observed in adolescents than in children, emphasizing a significant negative correlation between age and PA values.

Due to the mutation in the *DMD* gene, dystrophin is either not produced or produced at insufficient levels that decrease with the evolution of the disease. This protein plays an important structural role in muscle contraction, and its absence results in a notable alteration of the structure of the muscular membrane (26, 27). Therefore, the impaired integrity of the muscle membrane results in instability in the cell membrane and a subsequent decrease in the PA.

The BIVA is an autonomous procedure that is independent of predictive equations or models, allowing patients to be evaluated through direct measurements of the impedance vectors (19). BIVA studies have exhibited promising potential in evaluating hydration and nutrition in patients with one of several clinical conditions, such as renal diseases (28), critical illnesses (29), heart failure (30), obesity (31), Alzheimer’s disease (32), stroke (33), and cancer (34), as well as in athletes (35).

Considering the entire population of the study, approximately 87% of the subjects presented vectors outside the 50, 75, and 95% tolerance ellipses. Only one child exhibited a vector located in the 50% tolerance ellipse of the upper right quadrant, indicating normally hydrated and a good cell mass. The other participants presented vectors located outside of the tolerance ellipses, suggesting a lower cellularity and a tendency toward a smaller amount of muscle mass that correlated with the patient’s age.

The evident muscle mass loss observed in this population is caused by intense muscular atrophy due to the disease. Thus, nutritional status plays a key role in the quality of life and survival of patients with DMD (8). Low PA values and low cellularity levels are associated with morbidity and mortality in patients with some diseases (36), and these parameters may also be associated with a low nutritional prognosis for patients with DMD.

The identification of prognostic factors for patients with DMD is important for the clinical management of this disease. PA is being highlighted as a useful tool for evaluating patient prognosis under different clinical conditions (12, 23). PA is able to indicate the nutritional prognosis and can act as a predictor of survival in critically ill patients, as well as in patients with cirrhosis, cancer, nephropathies, and sepsis (11, 23, 37).

The strengths of our study include the use of unedited tools for nutritional assessment of patients with DMD. Our sampling process was limited because it was based on nonprobability. Other limitations of our study are the low frequency of DMD and the wide variation in the age of diagnosis of our sample. The generalizability of our findings will need to be assessed in prospective studies with more representative samples.

## Conclusions

Based on the evidence, patients with DMD had lower levels of body cell mass and hydration compared with the healthy population described in the literature. This evidence points to bioimpedance parameters as useful tools for the nutritional evaluation and clinical management of patients with DMD. PA and BIVA are easily accessible, low-cost procedures that display good reproducibility. Further studies with prospective design and comparing the results obtained by BIA devices with a gold standard method, such as dual-energy X-ray absorptiometry, are needed to validate these assessment methods in patients with DMD.

## Authors’ contributions

All authors were involved in the study design and collaborated to write this article. KMV, CXA, ECG, and MMGDL collected and analyzed the data, KMV, RJAM, MMGDL, and SHLV interpreted the data, SHLV and JBN coordinated the research, and KMV was primarily responsible for the final content.
